# Cumulative Success Rate of Short and Ultrashort Implants Supporting Single Crowns in the Posterior Maxilla: A 3-Year Retrospective Study

**DOI:** 10.1155/2017/8434281

**Published:** 2017-07-02

**Authors:** Giorgio Lombardo, Jacopo Pighi, Mauro Marincola, Giovanni Corrocher, Miguel Simancas-Pallares, Pier Francesco Nocini

**Affiliations:** ^1^School of Dentistry, Department of Surgery, Dentistry, Paediatrics and Gynaecology (DIPSCOMI), University of Verona, Verona, Italy; ^2^Dental Implant Unit, University of Cartagena, Cartagena, Colombia; ^3^Research Department, Faculty of Dentistry, University of Cartagena, Cartagena, Colombia

## Abstract

**Aim:**

To determine cumulative success rate (CSR) of short and ultrashort implants in the posterior maxilla restored with single crowns.

**Patients and Methods:**

We performed a retrospective study in 65 patients with 139 implants. 46 were ultrashort and 93 short. Implants were placed with a staged approach and restored with single crowns. Success rate, clinical and radiographic outcomes, and crown-to-implant ratio (CIR) were assessed after three years. Statistical analysis was performed by descriptive and inferential statistics. A log-binomial regression model where the main outcome was implant success was achieved. Coefficients and 95% confidence intervals were reported. Analyses were performed with Stata 13.2 for Windows.

**Results:**

61.54% of patients were female and mean overall age was 51.9 ± 11.08 years old. Overall CSR was 97.1% (95% CI: 92.4–98.9): 97.9 and 95.1% for short and ultrashort, respectively (*P* value: 0.33). Four implants failed. Covariates were not associated with CSR (*P* value > 0.05). Regression model showed coefficients correlated with implant success for ultrashort implants (0.87) and most of covariates but none were statistically significant (*P* values > 0.05).

**Conclusions:**

Our results suggest that short and ultrashort implants may be successfully placed and restored with single crowns in the resorbed maxillary molar region.

## 1. Introduction

The partially edentulous posterior maxilla bone quality is often poorly characterized by large marrow spaces and reduced both vertical and horizontal bone volumes due to the severe atrophy, increased sinus pneumatization, and also iatrogenic prosthesis. Patients with extremely atrophic upper posterior maxilla require major surgical sinus lift procedures [1] or even zygomatic implants to be successfully restored and then recover their oral function [2–5]. These options are clinically challenging, because of the increased patient morbidity and also the greater chance of intra- and postoperative complications [6, 7]. Likewise, the development of innovative implant designs and surface textures in cases of intermediate atrophy suggests the use of short implants as minimally invasive treatment options in these cases [8].

The definition of a short implant in scientific literature has been a historical debate. At first, “short” implants were defined as those with <11 mm in length [9, 10], 10 mm [11, 12], 8 mm [13], and 6 mm [14], and ultimately “extra-short” implants were defined as those with a ≤5 mm intrabony length [15]. However, the most recent European Consensus Conference on short, angulated, and diameter-reduced implants defined short implants as those with ≤8 mm in length and ≥3.75 mm in diameter, standard implants as those >8 mm in length and ≥3.75 mm in diameter, and ultrashort implants as those <6 mm in length [16]. Also they stated that short implants are used primarily to avoid bone augmentation procedures and they are applicable if vertical bone volume is limited by other anatomical structures such as maxillary sinus or the mandibular canal, but there is sufficient alveolar ridge width to use ≥3.75 mm diameter implants [16].

Short implants were historically associated with lower survival rates and with unpredictable long-term outcomes [17–20], but, currently due to their design improvement, scientific evidence suggests that short implants (>6 but ≤8 mm) have similar survival rates compared to standard implants (>8 mm) [15]. Splinted restorations were highly recommendable in the posterior area of the jaw in order to avoid unfavorable strains over the prosthesis [21], but further studies showed the success of nonsplinted short implants supporting single restorations, offering a comfortable prosthetic approach including better emergence profiles and oral hygiene access compared to other fixed partial prostheses options [22].

Several types of connections between the implant and its prosthetic abutment are commercially available. Screw-retained hexagonal (internal and external) or locking-taper have been subjected to research in the past [23]. Screw-retained systems exhibits greater rate of complications due to instability at the implant-abutment interface (IAI), poor accuracy of thread coupling, and the presence of microgap allowing microbial colonization at the IAI leading to higher rates of biological complications. To deal with this, the locking-taper connection was introduced. It is defined as a tapered connection with an angle connection <1.5 degrees on both components [24]. Major advantages of the locking-taper connection include increased mechanical stability with no micromovements or microgaps at the IAI, thus leading to fewer rates of biological and prosthetic complications. Numerous studies have shown the high survival rates of dental implants with this type of connection [23, 25, 26].

To the best of our knowledge most of these studies focused mainly on 8 mm length implant clinical outcomes, but the scientific evidence for 5 or 6 mm length implants supporting single crowns in the posterior jaw is scarce, thus leaving no clinical recommendations at this time for its clinical usage. So our aim was to determine cumulative success rates of 5 and 6 mm length implants in the posterior jaw restored with single crowns.

## 2. Materials and Methods

### 2.1. Study Design and Sample

We performed a retrospective study in 65 patients who had at least one 5, 6, or 8 mm length Bicon™ dental implant (Bicon Dental Implants, Boston, MA, USA) placed between January 2012 and December 2013 at the University of Verona Dental Clinic. One hundred thirty-nine dental implants were placed overall. Sample was selected by a convenience sampling according to the inclusion criteria described below.

### 2.2. Inclusion Criteria

Patients with ASA I or II status who voluntarily agreed to participate, aged > 18 years old, being partially edentulous in the posterior area of the maxilla, with a residual ridge that allowed insertion of ≤8 mm length implants, with 3 months of healing after tooth extraction and having at least one 8, 6, or 5 mm Bicon implant in length, and restored with single crowns with at least 3 years of function were included. Amongst all of the 139 implants, 52 were 8 mm, 46 were 6 mm, and 41 were 5 mm in length.

The study was conducted in accordance with the Helsinki statement, and all patients signed a written informed consent form. Also the University of Verona Institutional Review Board approved the protocol.

### 2.3. Preoperative Steps

Before implant placement, all patients received clinical examinations regarding periodontal diseases, caries, and soft tissue status and, if needed, dentate subjects were periodontally treated in order to obtain good oral health before implant placement. Also complete radiographic evaluation including panoramic and periapical radiographs with parallelism technique was obtained. When more than one implant was needed, surgical templates were delivered. All of the patients were prescribed Amoxicillin plus Clavulanate (Augmentin, GlaxoSmithKline SpA, Verona, Italy) one hour before the implant placement to prevent systemic or local infections.

### 2.4. Surgical Procedure

Local infiltrative anesthesia was used. 2% Xylocaine (Dentsply Pharmaceutical, York, PA, USA) was used to complete the surgical procedure.

Intrasulcular incisions were performed by using a N°15 blade in a Bard-Parker scalpel. Full thickness flap was obtained in the area and then this surgical protocol was followed for implant placement; we began with pilot (2 mm diameter) drilling to achieve cortical perforation. Initial pilot drilling length (3-4 mm) was determined upon residual bone height (RBH) measurement. This high-speed drill (1100 Revolutions Per Minute (RPMs)) was used with external saline irrigation and had a cutting edge at the apical portion. RBH aimed to determine also implant selection, but final pilot drilling length was calculated by adding 3 mm to the selected implant length. Once pilot drilling was performed, a periapical X-ray was obtained in order to control vertical and horizontal positions with regard to the adjacent anatomical structures.

The following steps were achieved with latch reamers (LRs) at 50 RPMs without external irrigation. LRs were used to widen the osteotomy, but length was always set at the computed final drilling length (by adding 3 mm to the desired implant length). LRs are designed with a 0.5 mm diameter progressive increase and were used until the final implant diameter was reached. Due to the fact that the LRs did not need external irrigation and have low RPMs, we collected autogenous bone from the latch reaming process. This bone was stored in a Silicone Dappen Dish during the procedure. Then the selected implant (Bicon Dental Implants, Boston, MA, USA) was manually inserted into the osteotomy through the healing plug. Healing plug was carefully removed and then, with a seating tip mounted into a straight handle, we seated the implant into the osteotomy. Healing plug was cut ensuring that no sharp edges were present and could irritate soft tissue. Then we placed harvested bone over the implant shoulder.

Single suture with polyglycolid acid (Vicryl, ACE Surgical Supply Co., Brockton, MA, USA) was used to close the incisions. After implant insertion, immediate postoperative X-ray was performed. The patient received postoperative and homecare instructions as well as antibiotic and analgesic prescriptions to avoid infections and pain/swelling, respectively.

After a 4-to-6-month healing period, the implants were uncovered, temporary abutments were placed, flaps were readapted, and sutures were placed around the temporary abutments. After 3 weeks of soft tissue healing, definitive impressions were taken and within 2 weeks definitive ceramic or composite single crown restorations were delivered. At each recall appointment and when needed, occlusal adjustments were made and the prosthetic restorations were checked for loosening, chipping, or other prosthetic complications.

### 2.5. Implant System

**Figure 1:**
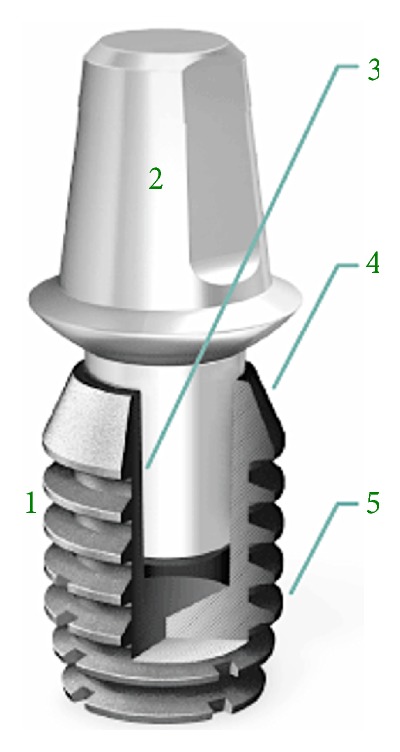
Schematic drawing of the Bicon dental implant system and its macrogeometric features. 1 represents the short root-plateau form implant body; 2 represents the abutment; 3 represents the 1.5° internal connection (locking-taper); 4 indicates the convergent crest module (sloping shoulder); and 5 represents the implant* plateaus*.

We used a locking-taper (Morse taper or Morse cone) dental implant system (Bicon Dental Implants, Boston, MA, United States) designed in 1985. Besides the aforementioned clinical advantages of a locking-taper connection with proven bacterial seal [27], this implant has a convergent crest module, platform switching, and a root-form plateau design (Figure 1). Regarding its surface, Integra-Ti™ (grit-blasted and acid-etched) and Integra-CP™ (Hydroxylapatite treated or covered by Hydroxyapatite) are commercially available.

### 2.6. Follow-Up Examination

**Figure 2:**
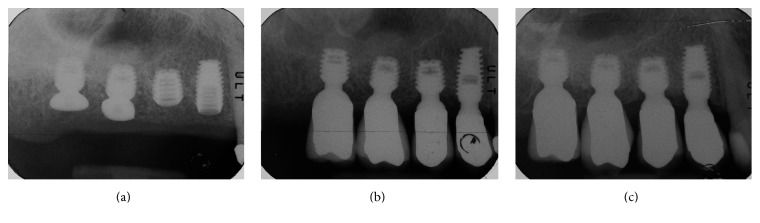
(a) Immediate postoperative radiography of premolar implants placed at the upper posterior maxilla (premolars); internal sinus lift cases were not included in this study. (b) Immediate X-ray obtained at crown insertion. (c) X-ray obtained at three-year follow-up showing the implant-restoration success.

After 3 years, patients were recalled for radiographic and clinical examinations. Peri-implant tissues and prostheses were also assessed.  Figure 2 depicts a case of two upper posterior-placed implants at the tooth numbers 14 and 15 with porcelain-fused-to-metal restorations. Number 14 was a 5.0 × 8.0 mm and number 15 a 5.0 × 5.0 mm implant, respectively.

### 2.7. Study Variables

All of the implants had the same diameter (5 mm), but three different lengths were included (5, 6, or 8 mm). The major predictor variable was implant length classified as short (S) (≤8 mm in length) or ultrashort (US) (<6 mm in length) according the proposed criteria of the European Consensus Conference on short, angulated, and diameter-reduced implants [16].

The main outcome was the cumulative success rate (CSR). Secondary variables (covariates) included the following: sex, age, smoking history, NSAIDs consumption, and clinical-related parameters such as implanted tooth type, history of periodontal disease on the treated site, and implant surface. Prosthetic-related covariates were type of restorative material and crown-to-implant ratio.

### 2.8. Crown-to-Implant Ratio (CIR) Determination

At first, crown height (in mm) was measured on the radiograph immediately after prosthetic loading as the most occlusal point to the implant-abutment interface (IAI) [28]. Then crown-to-implant ratios were calculated by dividing the digital length of the crown over the implant length and were dichotomized as >2 or <2 units.

Vertical distortion occurs equally in the crown and in the implant on the radiograph; and because the crown-to-implant ratio is not dependent on absolute values, the effect of vertical distortion on a ratio is then minimal [29].

### 2.9. Study Outcomes

#### 2.9.1. Primary Outcome: Cumulative Success Rate

Expressed as CSR was the primary outcome variable in our study. Failure was defined as the need of implant removal. Also implant failures were classified in two types: early (or initial) and late that occurred before and after implant loading (crown insertion), respectively.

#### 2.9.2. Secondary Outcomes: Biological and Prosthetic Complications

Biological complications included mucositis (swollen soft tissue and bleeding on probing without bone loss) and peri-implantitis (swollen tissues, bleeding on probing, bone loss, and peri-implant pocket depth > 5 mm) [30].

Prosthetic complications were considered as crown detachment, chipping, or material fracture. However, prosthesis failure was defined as the need to remake the crown due to fracture or loosening.

### 2.10. Statistical Analysis

We first performed univariate analysis through descriptive statistics. For qualitative variables, we computed proportions and 95% confidence intervals. However, to analyze quantitative data, we first tested normality assumptions by using the Shapiro-Wilks test. If normality criteria were met, we reported mean and standard deviation; otherwise median and interquartile range (IQR) were reported.

In bivariate analysis, we compared proportions using *χ*^2^ or Fisher's exact test, but, to compare means across groups, we used Student's* t*-test or the Mann–Whitney test, considering if normality assumption and homoscedasticity criteria were met (Levene's test).

Finally, for multivariate analysis, we created a generalized linear model (log-binomial regression) model where the outcome (*β*) was the implant success rate due to the high rate of the outcome [31]. Major predictors were defined a priori being those with biological plausibility. From this model we reported standardized coefficients and 95% confidence intervals. Marginal probability predictions were also estimated for each group of implant length (short or ultrashort). Statistical analysis was performed using Stata v.13.2 for Windows (StataCorp, College Station, TX, USA).

## 3. Results

Amongst the 65 patients, 61.5% were females. Overall mean age was 51.9 ± 11.08 years old. Most of the patients were nonsmokers (75.38%), ASA status II (52.31%), and non-NSAIDs consumers. Most of the implanted sites were located in the molar area, coated by the Integra-CP surface and restored using porcelain (porcelain-fused-to-metal (PFM) Technique).

When we analyzed sample distribution according to length definition (short or ultrashort), we only found statistical significance for patients having history of periodontal disease on the implanted site (58.46%), implanted tooth type (molars: 51.8%), and also type of restorative material (porcelain: 76.26%). Overall mean follow-up time was 32.69 ± 15.62 months.  Table 1 presents uni- and bivariate demographic and clinical-related outcomes.

Our overall cumulative success rate was 97.12% (95% CI: 92.49–98.92). Among the 139 implants, 4 implants failed: 3 due to peri-implantitis and the other one due to no osseointegration (early failure). When we analyzed failures according to implant length, implanted tooth type, restorative material, and also periodontal status before implantation, we found they were equally distributed amongst groups (*P* values > 0.05). Nonetheless, most of the failed implants were Integra-CP coated (4 implants) with crown-to-implant ratio > 2 (2 implants). Bivariate analysis for the cumulative success rate is presented in Table 2 showing no statistical significance of the aforementioned parameters with the cumulative success rates (*P* values > 0.05).

Table 3 shows the descriptive statistics for length definition distribution of the successes and failures according implanted tooth type, implant surface, restorative material, CIR, and also periodontal status before implantation.

Finally, we entered covariates into the log-binomial regression model and coefficients with 95% CI are presented in Table 4. Implant success increases with ultrashort implants in male patients with ASA status I, mostly consuming NSAIDs, and implants covered by Integra-CP. Regarding prosthetic covariates, success increases with crowns made by ceromer and with CIR > 2. On the other hand, failures increases in periodontally compromised patients. However, none of these parameters were statistically significant (*P* value > 0.05).

Probability prediction after regression indicated that overall probability of success for short and ultrashort implants are 96.24 and 94.39%, respectively. Finally, according to CIR (>2), probability of success for short and ultrashort implants was 95.64 and 93.51%, respectively.

## 4. Discussion

Placed implants in augmented bone in both mandible and maxilla simultaneously or after a staged 6-month period from lateral sinus floor elevation procedure were shown to provide high survival rates [32]. However, these associated procedures are highly invasive and often associated with a high rate of complications such as membrane perforation, sinusitis, and total or partial loss of the grafted material [33–37].

This led to an increase usage of short implants especially in the posterior area of the lower jaw. Also a large number of studies including systematic and narrative reviews suggest that short implants could be considered an alternative treatment to advanced bone augmentation techniques with significantly less complications rates and higher patient's satisfaction [38]. However, several of these reviews clustered short implants outcomes supporting different types of restoration, so the evidence about clinical outcomes of short implants supporting single crowns in the posterior maxilla is scarce [39, 40].

To the best of our knowledge, there is only one comprehensive systematic review aimed to evaluate the prognosis of the posterior area restoration with single crowns supported by short implants [41]. In this review, even when authors did not find differences between ≤6 and >7 but in ≤8 mm implants in length, they hypothesized that this might be due to the small sample of the 6 mm and 5 mm length implants included, and if, with a larger sample size of the 6 mm and 5 mm implants, the meta-regression analysis results should be different, finding statistically significant differences between implants ≤8 mm and >8 mm in length.

Recently, Lai et al. [42] in a 5–10-year study followed 231 short Straumann implants supporting single crowns. 110 implants were placed in the maxilla and found that the 6 and 8 mm length implants showed, respectively, a cumulative survival rate of 97 and 98.5%, with no differences in regard to the implanted jaw. Gulje et al. examined 41 patients randomly allocated to receive an 11 mm implant in combination with maxillary sinus floor elevation surgery or to receive a 6 mm implant without any grafting in the posterior maxilla. At the 12-month evaluation implant, survival was 100% in both groups [43]. Schincaglia et al. [44] and Bechara et al. [45], in two studies with similar designs, reported a cumulative survival rate of 100% for the 6 mm implants after 1 and 3 years, respectively. Even when we performed a mid-term (three years) follow-up, our results are also comparable to those at long-term (ten years) follow-up. Mangano et al., on a prospective clinical study including 215 short (8 mm) implants also supporting single crowns in the posterior region of the jaws, showed an implant-based cumulative survival rate of 98.5% [46], also highlighting the clinical applicability of short implants.

Our results show that overall cumulative success rates for short and ultrashort implants was 97.9% and 95.1%, respectively (*P* value = 0.58), thus being equivalent. These results are also consistent with previous evidence about short implants supporting single crowns in the posterior maxilla and, as a matter of fact, not only short but also ultrashort implants can support single crowns and remain as a successful treatment in the atrophic posterior maxilla, even with high C/I ratios (>2). It is important to note that the prevalence of crowns with CIR > 2 (47.4%) was higher than the previously published in the literature. Moreover when we analyzed success rates of short and ultrashort implants according to CIR, we did not find any statistical significance, thus suggesting that either short or ultrashort implants could be restored with single crowns having CIR > 2. Besides this, the multivariate analyses show the positive effect of CIR > 2 on the implant success rate (coefficient = 0.79). Even when our study only included dental implants restored by means of single crowns, our results are also comparable with those of Mangano et al., who showed a high CSR (97,2%), for standard (>10 mm) implants restored using fixed prosthesis with follow-up periods as high as 20 years [47].

Urdaneta et al. evaluated 326 short and ultrashort implants with the same implant design supporting single-tooth crowns with a mean C/IR of 1.6 (ranging from 0.79 to 4.95) and found that after 6 years (70.7 months) of follow-up a CIR up to 4.95 did not lead to an increased risk of implant failures, crown failures, or crown fractures [26]. Malchiodi et al., in a prospective study on 259 tapered truncated cone shaped implants with 5, 7, 9, or 12 mm in length, reported that 36% of the implants presented a C/I ratio >2, showing a CSR of 95.6% [48] being comparable to our results (96.9%). Anitua et al. [49], in a retrospective study with a mean follow-up of 28.9 months, reviewed the clinical outcomes of 128 short implants being mostly restored with bridges or splinted crowns and found a CSR of 100%. Only 42 out of the 128 implants (32.8%) had a >2 CIR. Recently, Mangano et al. [50], in a 5-year prospective study, followed 68 6.5 mm long implants in 51 patients. Twenty-nine out of the 65 implants (72%) were restored by means of single crowns. Twenty-five percent (17 out of 68) of the implants had at baseline CIR ≥ 2 and 3 failures were reported, all in the >2 CIR group.

Current scientific evidence demonstrates that implant design can play a determining role to allow higher clinical performance [51], and it is assumed to be particularly true for short implants. Moreover our results support these facts for ultrashort implants. Results from finite element analysis studies show that different implant bodies and abutment connection types may influence peri-implant bone stresses and abutment micromovement, determining the threshold values of tensile and shear stresses that cause resorption of cortical bone, thus affecting implant success rate [52].

Features like a reverse conical neck design, the locking-taper implant-abutment connection, and a plateau root form body are associated with low occlusal stress concentration on the buccal bone and limited harmful abutment micromovement inside the connection [52]. Furthermore, the locking-taper feature inhibits the bacterial leakage at the implant-abutment connection level [27], thus providing numerous benefits in terms of healing and osseointegration, leading to a better biomechanical fixation [53]. From a biomechanical standpoint, a locking-taper connection is mechanically more stable than external-hexagon or butt-joint implant-abutment connections. While the rate of biological and prosthetic complications related to screw-retained systems is high, locking-taper implants demonstrates minimizing all these problems. Also this type of implant-abutment connection can also withstand large lateral forces [54]. Thanks to the Morse taper principle, the high friction between the surfaces of two equal conical parts links them altogether. This phenomenon is known as “cold welding,” since both surfaces undergo a kind of interpenetration and fusion between their asperities as result of contact pressure. This means that both implant and abutment virtually create a single body; so compared to screw-retained systems, stress distribution is homogeneous through the unit [24].

All these facts that aim to elucidate the high CSR from our study are also well supported in scientific evidence from systematic reviews [55]. Implant placement in a subcrestal (submerged) fashion and the usage of an implant with convergent crest module, represented by the sloping shoulder geometry, enhance the platform switching (PS) to occur. This PS allows an increase in residual crestal alveolar bone volume around the neck of the an implant, repositions the papilla to a more esthetic and apposite level, reduces mechanical stress in the crestal alveolar bone area, and assists in enhancing the vascular supply to hard and soft tissue in case of reduced interdental space.

Even when our study was able to demonstrate the high cumulative success rate and low fate of biological complications, some limitations are evident such as the relatively small sample size, the mid-term follow-up, and imbalanced distribution across groups (short versus ultrashort). These factors might partially explain our nonstatistically significant associations presented here.

## 5. Conclusions

Results from our study suggest the adequate clinical performance of short and ultrashort implants. After 3 years of loading, the clinical applicability of these implants with locking-taper connection, sloping shoulder, and plateau-form which are supporting single crowns in the posterior maxilla is evident. Since we did not find any statistical difference between groups, even according to CIR, this is the first evidence to hypothesize that short and ultrashort implants are clinically equivalent and could be used either on premolar or on molar areas. However, in most cases where residual bone height in the molar area is limited, ultrashort implants are recommended for implant-supported restorations.

## Figures and Tables

**Table 1:** Overall placed implants according to studied covariates. Also distribution of placed implants according to implant length is presented in this table.

Variable	Overall		Short		Ultrashort		*P* value
	*n*	%	*n*	%	*n*	%	
*Sex*							
Female	40	61.54	26	65.00	14	56.00	0.46
Male	25	38.46	14	35.00	11	44.00	
*Age*	51.90 ± 11.08		51.07 ± 10.38		53.24 ± 12.22		0.44
*Smoking history*							
Yes	16	24.62	7	17.50	9	36.00	0.09
No	49	75.38	33	82.50	16	64.00	
*ASA status*							
I	31	47.69	21	52.50	10	40.00	0.32
II	34	52.31	19	47.50	15	60.00	
*NSAIDs consumption*							
Yes	5	7.69	2	5.00	3	12.00	0.36
No	60	92.31	38	95.00	22	88.00	
*History of periodontal disease*							
Yes	38	58.46	19	47.50	19	76.00	0.02^*∗*^
No	27	41.54	21	52.50	6	24.00	
*Implanted tooth type*							
Premolar	67	(48.20)	56	(57.14)	11	(26.83)	0.00^*∗*^
Molar	72	(51.80)	42	(42.86)	30	(73.17)	
*Implant surface*							
Integra-CP	117	87.31	80	84.21	37	94.87	0.29
HA-coated	12	8.96	10	10.53	2	8.96	
Integra-Ti	5	3.73	5	5.26	0	3.73	
*Restorative material*							
Ceromer	33	(23.74)	18	(18.37)	15	(36.59)	0.02^*∗*^
Porcelain	106	(76.26)	80	(81.63)	26	(63.41)	

^*∗*^Statistically significant differences between groups. Age is presented as mean ± standard deviation.

**Table 2:** Bivariate analysis of the success rate according to study included covariates.

Variable	Overall		*P* value
	Success	Failure	
	*n* (%)	*n* (%)	
*Implant length*	****		
Short	96 (97.96)	2 (2.04)	0.33
Ultrashort	39 (95.12)	2 (4.88)	
*Implanted tooth type*			
Premolar	65 (97.01)	2 (2.99)	1.00
Molar	70 (97.22)	2 (2.78)	
*Implant surface*			
Integra-CP	113 (96.58)	4 (3.42)	1.00
Hydroxyapatite (HA) coating	12 (100)	0 (0.00)	
Integra-Ti	5 (100)	0 (0.00)	
*Restorative material*			
Ceromer	31 (93.94)	2 (6.06)	0.23
Ceramic	104 (98.11)	2 (1.89)	
*Crown-to-implant ratio*			
<2 Units	71 (98.61)	1 (1.39)	0.60
>2 Units	63 (96.92)	2 (3.08)	
*Periodontal status before implantation*			
Periodontally compromised	36 (94.74)	2 (5.26)	1.00
Nonperiodontally compromised	25 (92.59)	2 (7.41)	

**Table 3:** Descriptive analysis of the success rate according to study groups.

Variable	Short		Ultrashort	
	Success	Failure	Success	Failure
	*n* (%)	*n* (%)	*n* (%)	*n* (%)
*Implanted tooth type*				
Premolar	55 (57.29)	1 (50.00)	10 (25.64)	1 (50.00)
Molar	41 (42.70)	1 (50.00)	29 (74.35)	1 (50.00)
*Implant surface*				
Integra-CP	78 (83.87)	2 (100)	35 (94.59)	2 (100)
HA-coated	10 (10.75)	0 (0.00)	2 (5.40)	0 (0.00)
Integra-Ti	5 (5.37)	0 (0.00)	0 (0.00)	0 (0.00)
*Restorative material*				
Ceromer	18 (18.75)	0 (0.00)	13 (33.33)	2 (100)
Ceramic	78 (81.25)	2 (100)	26 (66.66)	0 (0.00)
*Crown-to-implant ratio*				
<2 Units	66 (69.47)	1 (50.00)	5 (12.82)	0 (0.00)
>2 Units	29 (30.52)	1 (50.00)	34 (87.17)	1 (100)
*Periodontal status before implantation*				
Periodontally compromised	20 (52.63)	1 (50.00)	5 (21.73)	1 (50.00)
Nonperiodontally compromised	18 (47.36)	1 (50.00)	18 (78.26)	1 (50.00)

**Table 4:** Standardized coefficients derived from log-binomial regression and 95% confidence intervals for factors associated with success rate.

Variable	Estimates		*P* value
	Coefficients	95% CI	
*Implant length*			
Ultrashort	0.87	1.05–2.79	0.37
*Sex*			
Male	0.47	−1.42–2.36	0.62
*ASA status*			
I	0.09	−1.80–1.99	0.92
*NSAIDs consumption*			
Yes	1.38	−0.68–3.45	0.19
*Implant surface*			
Integra-CP	14.85	−4318.28–4348.00	0.99
HA-coated	−14.19	−3787.47–3759.08	
Integra-Ti	−13.88	−5171.07–5143.29	
*Restorative material*			
IACs (ceromer)	1.16	−0.75–3.08	0.23
*Crown-to-implant ratio*			
>2 units	0.79	−1.58–3.17	0.51
*Periodontal status before implantation*			
Periodontally compromised	−0.34	−2.23–1.55	0.72

## References

[1] Pjetursson B. E., Tan W. C., Zwahlen M., Lang N. P. (2008). A systematic review of the success of sinus floor elevation and survival of implants inserted in combination with sinus floor elevation: part I: lateral approach. *Journal of Clinical Periodontology*.

[2] Lombardo G., D'Agostino A., Trevisiol L. (2016). Clinical, microbiologic and radiologic assessment of soft and hard tissues surrounding zygomatic implants: a retrospective study. *Oral Surgery, Oral Medicine, Oral Pathology and Oral Radiology*.

[3] Nocini P. F., Trevisiol L., D’Agostino A., Zanette G., Favero V., Procacci P. (2016). Quadruple zygomatic implants supported rehabilitation in failed maxillary bone reconstruction. *Oral and Maxillofacial Surgery*.

[4] D'Agostino A., Pasquale P., Ferrari F., Trevisiol L., Francesco N. P. (2013). Zygoma implant-supported prosthetic rehabilitation of a patient after subtotal bilateral maxillectomy. *Journal of Craniofacial Surgery*.

[5] Nocini P. F., D'agostino A., Chiarini L., Trevisiol L., Procacci P. (2014). Simultaneous le fort I osteotomy and zygomatic implants placement with delayed prosthetic rehabilitation. *Journal of Craniofacial Surgery*.

[6] Boffano P., Forouzanfar T. (2014). Current concepts on complications associated with sinus augmentation procedures. *Journal of Craniofacial Surgery*.

[7] D'Agostino A., Trevisiol L., Favero V., Pessina M., Procacci P., Nocini P. F. (2016). Are zygomatic implants associated with maxillary sinusitis?. *Journal of Oral and Maxillofacial Surgery*.

[8] Alqutaibi A. Y., Altaib F. (2016). Short dental implant is considered as a reliable treatment option for patients with atrophic posterior maxilla. *Journal of Evidence-Based Dental Practice*.

[9] das Neves F., Fones D., Bernardes S., do Prado C., Neto A. (2006). Short implants-an analysis of longitudinal studies. *The International Journal of Oral & Maxillofacial Implants*.

[10] Strietzel F. P., Reichart P. A. (2007). Oral rehabilitation using Camlog screw-cylinder implants with a particle-blasted and acid-etched microstructured surface. Results from a prospective study with special consideration of short implants. *Clinical Oral Implants Research*.

[11] Morand M., Irinakis T. (2007). The challenge of implant therapy in the posterior maxilla: providing a rationale for the use of short implants.. *The Journal of oral implantology*.

[12] Tawil G., Younan R. (2003). Clinical evaluation of short, machined-surface implants followed for 12 to 92 months. *The International Journal of Oral & Maxillofacial Implants*.

[13] Renouard F., Nisand D. (2006). Impact of implant length and diameter on survival rates. *Clinical Oral Implants Research*.

[14] Srinivasan M., Vazquez L., Rieder P., Moraguez O., Bernard J.-P., Belser U. C. (2012). Efficacy and predictability of short dental implants (<8 mm): a critical appraisal of the recent literature. *International Journal of Oral and Maxillofacial Implants*.

[15] Nisand D., Renouard F. (2014). Short implant in limited bone volume. *Periodontology 2000*.

[16] Neugebauer J., Nickenig H., Zöller J. Update on short, angulated an diameter-reduced implants.

[17] Bahat O. (2000). Branemark system implants in the posterior maxilla: clinical study of 660 implants followed for 5 to 12 years. *International Journal of Oral and Maxillofacial Implants*.

[18] Winkler S., Morris H. F., Ochi S. (2000). Implant survival to 36 months as related to length and diameter. *Annals of periodontology*.

[19] Pierrisnard L., Renouard F., Renault P., Barquins M. (2003). Influence of implant length and bicortical anchorage on implant stres distribution. *Clinical Implant Dentistry and Related Research*.

[20] Weng D., Jacobson Z., Tarnow D., Hurzeler M. B., Faehn O., Sanavi F. (2003). A prospective multicenter clinical trial of 3i machined-surface implants: results after 6 years of follow-up. *The International Journal of Oral & Maxillofacial Implants*.

[21] Rangert B. R., Sullivan R. M., Jemt T. M. (1997). Load factor control for implants in the posterior partially edentulous segment. *International Journal of Oral and Maxillofacial Implants*.

[22] Solnit G. S., Schneider R. L. (1998). An alternative to splinting multiple implants: use of the ITI system. *Journal of Prosthodontics*.

[23] Demiralp K. O., Akbulut N., Kursun S., Argun D., Bagis N., Orhan K. (2015). Survival rate of short, locking taper implants with a plateau design: a 5-year retrospective study. *BioMed Research International*.

[24] Sannino G., Barlattani A. (2013). Mechanical evaluation of an implant-abutment self-locking taper connection: finite element analysis and experimental tests. *The International Journal of Oral & Maxillofacial Implants*.

[25] Mangano F., Macchi A., Caprioglio A., Sammons R. L., Piattelli A., Mangano C. (2014). Survival and complication rates of fixed restorations supported by locking-taper implants: a prospective study with 1 to 10 years of follow-up. *Journal of Prosthodontics*.

[26] Urdaneta R. A., Daher S., Leary J., Emanuel K. M., Chuang S. K. (2012). The survival of ultrashort locking-taper implants. *The International Journal of Oral & Maxillofacial Implants*.

[27] Dibart S., Warbington M., Su M. F., Skobe Z. (2005). In vitro evaluation of the implant-abutment bacterial seal: the locking taper system. *International Journal of Oral and Maxillofacial Implants*.

[28] Blanes R. J., Bernard J. P., Blanes Z. M., Belser U. C. (2007). A 10-year prospective study of ITI dental implants placed in the posterior region. II: influence of the crown-to-implant ratio and different prosthetic treatment modalities on crestal bone loss. *Clinical Oral Implants Research*.

[29] Birdi H., Schulte J., Kovacs A., Weed M., Chuang S.-K. (2010). Crown-to-implant ratios of short-length implants. *The Journal of Oral Implantology*.

[30] Sanz M., Chapple I. L., Working Group 4 of the VEWoP (2012). Clinical research on peri-implant diseases: consensus report of working group 4. *Journal of Clinical Periodontology*.

[31] Skov T., Deddens J., Petersen M. R., Endahl L. (1998). Prevalence proportion ratios: estimation and hypothesis testing. *International Journal of Epidemiology*.

[32] Aloy-Prosper A., Peñarrocha-Oltra D., Penarrocha-Diago M., Penarrocha-Diago M. (2015). The outcome of intraoral onlay block bone grafts on alveolar ridge augmentations: a systematic review. *Medicina Oral, Patologia Oral y Cirugia Bucal*.

[33] Tan W. C., Lang N. P., Zwahlen M., Pjetursson B. E. (2008). A systematic review of the success of sinus floor elevation and survival of implants inserted in combination with sinus floor elevation Part II: Transalveolar technique. *Journal of Clinical Periodontology*.

[34] Chan H.-L., Wang H.-L. (2011). Sinus pathology and anatomy in relation to complications in lateral window sinus augmentation. *Implant Dentistry*.

[35] Lee H.-W., Lin W.-S., Morton D. (2013). A retrospective study of complications associated with 100 consecutive maxillary sinus augmentations via the lateral window approach. *International Journal of Oral and Maxillofacial Implants*.

[36] Camps-Font O., Burgueno-Barris G., Figueiredo R., Jung R. E., Gay-Escoda C., Valmaseda-Castellon E. (2016). Interventions for dental implant placement in atrophic edentulous mandibles: vertical bone augmentation and alternative treatments. a meta-analysis of randomized clinical trials. *Journal of Periodontology*.

[37] Draenert F. G., Kammerer P. W., Berthold M., Neff A. (2016). Complications with allogeneic, cancellous bone blocks in vertical alveolar ridge augmentation: prospective clinical case study and review of the literature. *Oral Surgery, Oral Medicine, Oral Pathology and Oral Radiology*.

[38] Pommer B., Mailath-Pokorny G., Haas R., Busenlechner D., Furhauser R., Watzek G. (2014). Patients' preferences towards minimally invasive treatment alternatives for implant rehabilitation of edentulous jaws. *European journal of oral implantology*.

[39] Thoma D. S., Cha J., Jung U. (2017). Treatment concepts for the posterior maxilla and mandible: short implants versus long implants in augmented bone. *Journal of Periodontal & Implant Science*.

[40] Lemos C. A. A., Ferro-Alves M. L., Okamoto R., Mendonça M. R., Pellizzer E. P. (2016). Short dental implants versus standard dental implants placed in the posterior jaws: a systematic review and meta-analysis. *Journal of Dentistry*.

[41] Mezzomo L. A., Miller R., Triches D., Alonso F., Shinkai R. S. A. (2014). Meta-analysis of single crowns supported by short (<10 mm) implants in the posterior region. *Journal of Clinical Periodontology*.

[42] Lai H.-C., Si M.-S., Zhuang L.-F., Shen H., Liu Y.-L., Wismeijer D. (2013). Long-term outcomes of short dental implants supporting single crowns in posterior region: a clinical retrospective study of 5–10 years. *Clinical Oral Implants Research*.

[43] Gulje F. L., Raghoebar G. M., Vissink A., Meijer H. J. (2014). Single crowns in the resorbed posterior maxilla supported by either 6-mm implants or by 11-mm implants combined with sinus floor elevation surgery: a 1-year randomised controlled trial. *European Journal of Oral Implantology*.

[44] Schincaglia G. P., Thoma D. S., Haas R. (2015). Randomized controlled multicenter study comparing short dental implants (6 mm) versus longer dental implants (11–15 mm) in combination with sinus floor elevation procedures. Part 2: Clinical and radiographic outcomes at 1 year of loading. *Journal of Clinical Periodontology*.

[45] Bechara S., Kubilius R., Veronesi G., Pires J. T., Shibli J. A., Mangano F. G. (2016). Short (6-mm) dental implants versus sinus floor elevation and placement of longer (≥10-mm) dental implants: a randomized controlled trial with a 3-year follow-up. *Clinical Oral Implants Research*.

[46] Mangano F. G., Shibli J. A., Sammons R. L., Iaculli F., Piattelli A., Mangano C. (2014). Short (8-mm) locking-taper implants supporting single crowns in posterior region: a prospective clinical study with 1-to 10-years of follow-up. *Clinical Oral Implants Research*.

[47] Mangano C., Iaculli F., Piattelli A., Mangano F. (2015). Fixed restorations supported by Morse-taper connection implants: a retrospective clinical study with 10–20 years of follow-up. *Clinical Oral Implants Research*.

[48] Malchiodi L., Cucchi A., Ghensi P., Consonni D., Nocini P. F. (2014). Influence of crown-implant ratio on implant success rates and crestal bone levels: a 36-month follow-up prospective study. *Clinical Oral Implants Research*.

[49] Anitua E., Pinas L., Orive G. (2015). Retrospective study of short and extra-short implants placed in posterior regions: influence of crown-to-implant ratio on marginal bone loss. *Clinical Implant Dentistry and Related Research*.

[50] Mangano F., Frezzato I., Frezzato A., Veronesi G., Mortellaro C., Mangano C. (2016). The effect of crown-to-implant ratio on the clinical performance of extra-short locking-taper implants. *Journal of Craniofacial Surgery*.

[51] Lee K.-J., Kim Y.-G., Park J.-W., Lee J.-M., Suh J.-Y. (2012). Influence of crown-to-implant ratio on periimplant marginal bone loss in the posterior region: a five-year retrospective study. *Journal of Periodontal and Implant Science*.

[52] Yamanishi Y., Yamaguchi S., Imazato S., Nakano T., Yatani H. (2012). Influences of implant neck design and implant-abutment joint type on peri-implant bone stress and abutment micromovement: three-dimensional finite element analysis. *Dental Materials*.

[53] Coelho P. G., Suzuki M., Guimaraes M. V. M. (2010). Early bone healing around different implant bulk designs and surgical techniques: a study in dogs. *Clinical Implant Dentistry and Related Research*.

[54] Gotfredsen K., Berglundh T., Lindhe J. (2001). Bone reactions adjacent to titanium implants subjected to static load: a study in the dog. *Clinical Oral Implants Research*.

[55] Schmitt C. M., Nogueira-Filho G., Tenenbaum H. C. (2014). Performance of conical abutment (Morse Taper) connection implants: a systematic review. *Journal of Biomedical Materials Research A*.

